# Downregulation of HDAC1 Is Involved in the Cardiomyocyte Differentiation from Mesenchymal Stem Cells in a Myocardial Microenvironment

**DOI:** 10.1371/journal.pone.0093222

**Published:** 2014-04-01

**Authors:** Dong-feng Lu, Yan Yao, Zi-zhuo Su, Zhao-hua Zeng, Xiao-wen Xing, Zhi-yu He, Chunxiang Zhang

**Affiliations:** 1 Department of Cardiology and Sino-US Cardiovascular Research Center, The First Affiliated Hospital of Guangzhou Medical University, Guangzhou, Guangdong, China; 2 Department of Pharmacology and Cardiovascular Research Center, Rush Medical College, Rush University, Chicago, Illinois, United States of America; Georgia Regents University, United States of America

## Abstract

Under myocardial microenvironment, bone marrow-derived mesenchymal stem cells (MSCs) can transdifferentiate into cardiomyocytes (CMs). However, the role of histone deacetylase 1 (HDAC1) in this directed differentiation process remains unclear. The current study is to determine the acetylation regulatory mechanisms that may be involved in the directed CM differentiation from MSCs. MSCs isolated from male Sprague-Dawley (SD) rats were marked with Ad-EGFP and co-cultured with CMs. Flow cytometry was used to sort EGFP-positive (EGFP+) MSCs from the co-culture system. Then, the expression of cardiac troponin T (cTnT) in these MSCs was detected by immunofluorescence assay. In addition, HDAC1 levels at different co-culture times were measured by quantitative real-time polymerase chain reaction (QT-PCR) and Western blotting. At 4 days after co-culture with CMs, the MSCs began to expression detectable levels of cTnT. The expression of HDAC1 in CMs was much lower than that in MSCs. After co-culture with CMs, the expression of HDAC1 in MSCs was significantly decreased in a time dependent manner. In addition, our recent study has also identified that knockdown of the HDAC1 could promote the directed differentiation of MSCs into CMs. The results suggest that HDAC1 has a negative correlation with cardiac cell differentiation from MSCs under a myocardial microenvironment. HDAC1 might play an important role in the directed differentiation of MSCs into CMs in heart.

## Introduction

Bone marrow-derived mesenchymal stem cells (MSCs) exhibit strong capabilities of self-renewal and tri-lineage differentiation and are suitable for autologous stem cell therapy in many diseases including ischemic heart disease. Indeed, MSCs are able to differentiate into tissue-specific mature cells in their targeted organs, in which the tissue-specific microenvironment plays important roles. However, the detailed molecular mechanisms responsible for the microenvironment-mediated MSC directed differentiation are still unclear [1]. Recent studies have revealed that epigenetic changes in DNA methylation and chromatin structure critical in the determination of lineage-specific differentiation of MSCs [2]. In this respect, histone deacetylase inhibitors have been reported to have strong effects on MSC differentiation [3]. Moreover, epigenetic modifications may provide “bridges” that link environmental signals to the responses in genes that could induce stem cell differentiation [4], [5], [6], [7]. For example, the epigenetic modification of histone acetylation has been identified to play an important role in the transdifferentiation processes of adult stem cells.

It is established that MSCs are able to differentiate into cardiac cells under heart-specific microenvironment [8]. However, the role of histone acetylation in cardiac microenvironment-induced differentiation of MSCs into CMs is still unclear. In the current study, in order to determine the potential role of histone acetylation in directed cardiac cell differentiation, the expression of histone deacetylase 1 (HDAC1) was determined in MSCs during their transdifferentiation into CMs in an *in vitro* model of the myocardial microenvironment.

## Materials and Methods

### 1. Animals

Four to six-week-old Male Sprague-Dawley (SD) rats and 1 to 2 day-old SD rats were from Sun Yat-sen University. All animal protocols were approved by the Institutional Animal Care and Use Committee at the Guangzhou Medical University and were consistent with the Guide for the Care and Use of Laboratory Animals (NIH publication 85–23, revised 1985).

### 2. The isolation, culture, and identification of bone marrow-derived MSCs

The isolation and culture of MSCs from 4 to 6-week-old male SD rats was accomplished using the whole bone marrow adherence method described previously [9]. Briefly, after sacrification, rat femora and tibiae were rapidly stripped. A 5 ml needle-syringe loaded with an appropriate volume of complete MSC culture medium (Dulbecco's modified Eagle's medium/Ham's F12 nutrient mixture (DMEM/F12) with 10% FBS, 100 U/ml penicillin, and 100 U/ml streptomycin) was inserted into the metaphysis of each bone to flush bone marrow cells. The cells were centrifuged at 1000 rpm for 10 minutes, resuspended in complete medium, and cultured. The culture medium was changed every 2–3 days. After reaching 80% of confluence, cultured adherent cells were passaged with a 0.25% trypsin solution. The percentages of cells that were positive for the MSC surface markers of CD34, CD45, CD29, CD44, and CD90 (Santa Cruz) were analysed by flow cytometry. Cultured MSCs at passage three to five (P3 to P5) were used for the experiments.

### 3. The transfection of MSCs with adenoviral vector encoding enhanced green fluorescent protein (Ad-EGFP)

Cultured MSCs were seeded in six-well plates at a density of 1×10^5^/ml. After 48 hours, Ad-EGFP was added to the culture wells at the following multiplicities of infection (MOI): 0, 10, 50, 100, 200, and 400. Cellular EGFP was observed after 72 hours' infection by fluorescence microscopy. Subsequently, cells were trypsinised, and the percentage of EGFP-positive (EGFP+) MSCs was assessed by flow cytometry.

### 4. The isolation and culture of neonatal CMs

The isolation and culture of neonatal CMs was performed as described in our previous studies [10], [11]. Briefly, myocardial tissues from 1 to 2-day old rats were cut into 1 mm^3^ cubes with ophthalmic scissors; 5–8 volumes of 0.25% trypsin was added to each tissue sample, and these samples were digested in a shaking water bath at a constant temperature of 37°C until the tissue cubes became white. After digestion, the cell suspension was passed through a 200-mesh cell sieve and collected by centrifugation at 1500 rpm for 5 minutes. Cells were resuspended in an appropriate quantity of CM complete medium (DMEM/F12 with 15% FBS, 100 U/ml penicillin, 100 U/ml streptomycin, and 0.1 mM BrdU). After purification, the CMs were seeded in 100 mm culture dishes at a density of 5×10^6^/ml. The medium was changed at 48 hours after seeding and every 2–3 days thereafter. After 5 days, the cultured cells were used for the experiments.

### 5. The creation of the co-culture system, designation of experimental groups, and separation of EGFP+ MSCs

MSCs were incubated in complete culture medium containing mitomycin C (at a final concentration of 5 μg/ml) for 2 hours to inhibit mitosis. Subsequently, these cells were washed at least six times with phosphate-buffered saline (PBS) and transfected with Ad-EGFP. At 72 hours after transfection, the cells were trypsinised and added into CM culture dishes at a 1∶1 ratio. The culture medium was changed at 48 hours after co-culture and every 2–3 days thereafter. Six experimental groups were designed based on culture conditions and culture times, which included the MSC group (the control group), the CM group, and 4 co-cultured groups that had been co-cultured for 3, 6, 9, or 12 days. Cells (>1×10^7^) were collected from each group and sorted by flow cytometry to identify MSCs with EGFP (EGFP+ MSCs).

### 6. Immunofluorescence staining

CMs or EGFP+MSCs from each experimental group were fixed on slides with 4% paraformaldehyde (PFA) for 20 minutes, permeabilised with 0.5% Triton X-100 for 20 minutes, and treated with 3% H_2_O_2_ for 15 minutes. The cell samples were blocked with 5% bovine serum albumin (BSA) for 30 minutes at 37°C and incubated overnight at 4°C with either anti-cardiac troponin T (cTnT) antibody (1∶400, Abcam) or PBS (as a negative control). The cell samples were then incubated with the secondary antibody CY3-IgG (1∶100) for 45 minutes at 37°C and with DAPI (5 μg/ml) at room temperature for 15 minutes. Fluorescence microscopy was used to detect cTnT expression in these cells.

### 7. Quantitative real-time polymerase chain reaction (QT-PCR)

TRIzol was used to extract total RNA samples from cells. The TaKaRa transcription kit was used for the reverse transcription of these samples. In this reaction, a PCR thermocycler was used to incubate the samples at 37°C for 15 minutes and then at 85°C for 5 seconds. The TaKaRa SYBR Green I fluorescence quantitative PCR kit was employed for the QT-PCR amplification of the resulting cDNA samples, using glyceraldehyde 3-phosphate dehydrogenase (GAPDH) as an internal control. The amplification included the following three reaction stages: stage I (initial denaturation), which had an incubation at 95°C for 30 seconds; stage II (40 cycles of PCR amplification), which had 40 cycles of incubation at 95°C for 5 seconds followed by an incubation at 60°C for 20 seconds; and stage III (melting curve analysis), which had incubation at 95°C for 15 seconds, 60°C for 1 minute, and 95°C for 15 seconds. The PCR primers were designed using the Primer 6.0 software package and synthesised by TaKaRa Biotechnology. In particular, the following specific primers were synthesised: HDAC1-F, TCA CCG AAT CCG AAT GAC TCA TAA; HDAC1-R, CTG GGC GAA TAG AAC GCA AGA; GAPDH-F, GGC ACA GTC AAG GCT GAG AAT G; and GAPDH-R, ATG GTG GTG AAG ACG CCA GTA. Based on the amplification results, the 2^−ΔΔCT^ method was used to calculate the relative levels of mRNAs.

### 8. Western blotting

At least 1×10^7^ cells were collected for the extraction of nucleoproteins, which was performed using the DBI nucleoprotein extraction kit. Protein concentrations were determined using the bicinchoninic acid (BCA) assay. A sample of 110 μg of nucleoproteins was separated on a 10% sodium dodecyl sulphate-polyacrylamide gel electrophoresis (SDS-PAGE) gel (100 V, 4 hours) and subsequently electrotransferred onto polyvinylidene difluoride (PVDF) membranes at 4°C (300 mA, 2.5 hours). After blocking with non-fat dry milk (5%) for 1.5 hours, these membranes were incubated overnight at 4°C with anti-rat HDAC1 (1∶1000, Abcam) and GAPDH (1∶500, Santa Cruz) antibodies. The membranes were then incubated with horseradish peroxidase (HRP)-labelled secondary antibody (1∶3000) for 2 hours and treated with an enhanced chemiluminescence (ECL) solution. Images of the membranes were digitally developed over a 5-minute exposure in a darkroom. For each experimental group, the relative expression level of a target protein was quantified by dividing the greyscale value of the target protein band by the greyscale value of the band corresponding to GAPDH.

### 9. Statistical analysis

Statistical analyses were performed using the SPSS 13.0 statistical software package, and the data were expressed as means ± standard deviation. Comparisons between two groups were performed using the independent samples t-test, and comparisons among multiple groups were performed using the one-way analyses of variance; P<0.05 was regarded as statistically significant.

## Results

### 1. The morphological characteristics and identification of MSCs in culture

**Figure 1:**
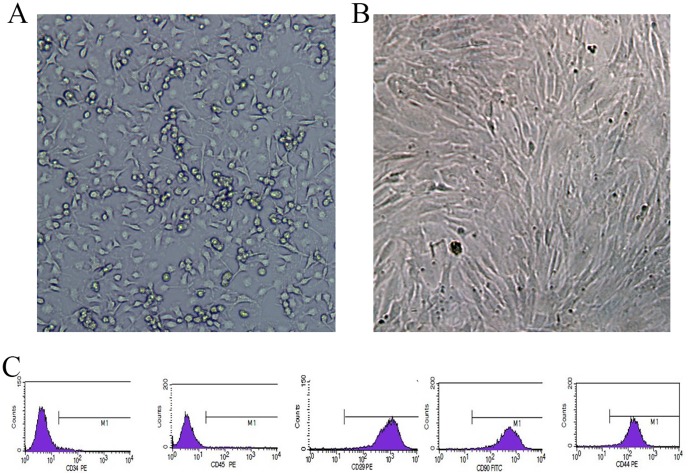
Morphological observations surface marker detection of bone marrow-derived MSCs. A) Primary MSCs cultured for 24 hours; B) MSCs at passage 3; C) The relative levels of makers of CD34, CD45, CD29, CD44, and CD90 in MSCs determined by flow cytometry.

**Figure 2:**
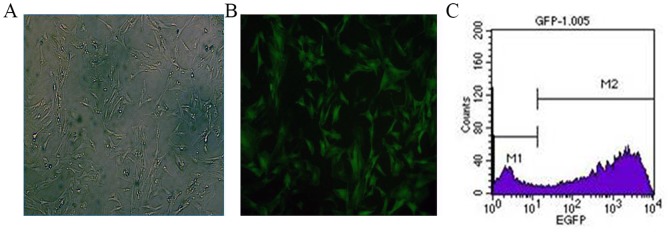
MSCs at 72-EGFP. A) Representative image of MSCs under Inverted microscopy; B) Representative image of MSCs under fluorescence microscopy; C) Flow cytometry assessments of transfection efficiency.

After 48–72 hours of primary culture, MSCs exhibited a fusiform morphology with the formation of clonal colonies. After 9–10 days of culture, these colonies had merged to form a confluent monolayer of cells at the bottom of the culture dish. After passaging, cells exhibited a fibroblast-like shape and reached 80% confluence after 3–4 days of culture. As the number of passages increased, cell morphologies became more consistent, and the MSCs exhibited spiral-shaped arrangements or patterns reminiscent of schools of fish (Figure 1A and 1B). Flow cytometric analyses of MSCs demonstrated that these cells did not express the hematopoietic markers CD34 (3.04%) and CD45 (1.14%) but strongly expressed various stromal and mesenchymal cell surface markers, including CD29 (99.86%), CD44 (99.28%), and CD90 (99.74%) (Figure 2C).

### 2. Ad-EGFP transfection efficiency

EGFP expression began to be observed in MSCs at 24 hours after Ad-EGFP transfection. The EGFP expression reached peak levels at 72 hours after this transfection and began to diminish at 1 week after transfection. At MOIs of 10 to 100, the transfection efficiency was lower than 60%. In contrast, at an MOI of 400, a transfection efficiency of up to 80–85% was observed. However, the cytopathic effect (CPE) was induced and significant MSC death occurred at 400 MOI. At an MOI of 200, a transfection efficiency of 80% could be achieved with only a few dead cells and no significant morphological changes (Figure 2).

### 3. The morphological characteristics and cTnT expression of CMs cultured *in vitro*

**Figure 3:**
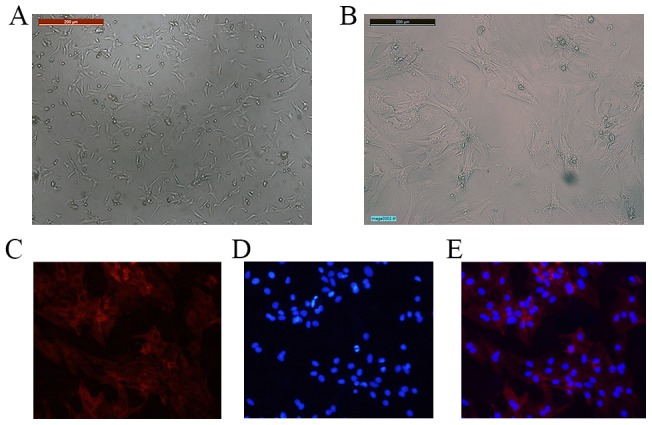
Morphological images of CMs and their expression of cTnT. A) CMs cultured for 24 hours; B) CMs cultured for 5 days; D) cTnT staining (red); E. Nuclei staining with DAPI (blue); C) Merged images of D&E.

Spontaneous contraction of isolated CMs was observed at 24 hours of culture. After 72 hours of culture, the CMs exhibited triangular or flat irregular shapes and began to form the clustered distributions, in which cell-cell contacts were observed. After 5 days of culture, the CMs appeared to be fully extended in shape, and cellular volumes increased (Figure 3A). At this time, consistent spontaneous pulsation of the CMs was observed at a frequency of 80–120 contractions/minute. Immunofluorescence signals of cTnT were identified in these cultured CMs (Figure 3B).

### 4. The co-culture of EGFP+MSCs with CMs and the expression of cTnT in co-cultured cells

**Figure 4:**
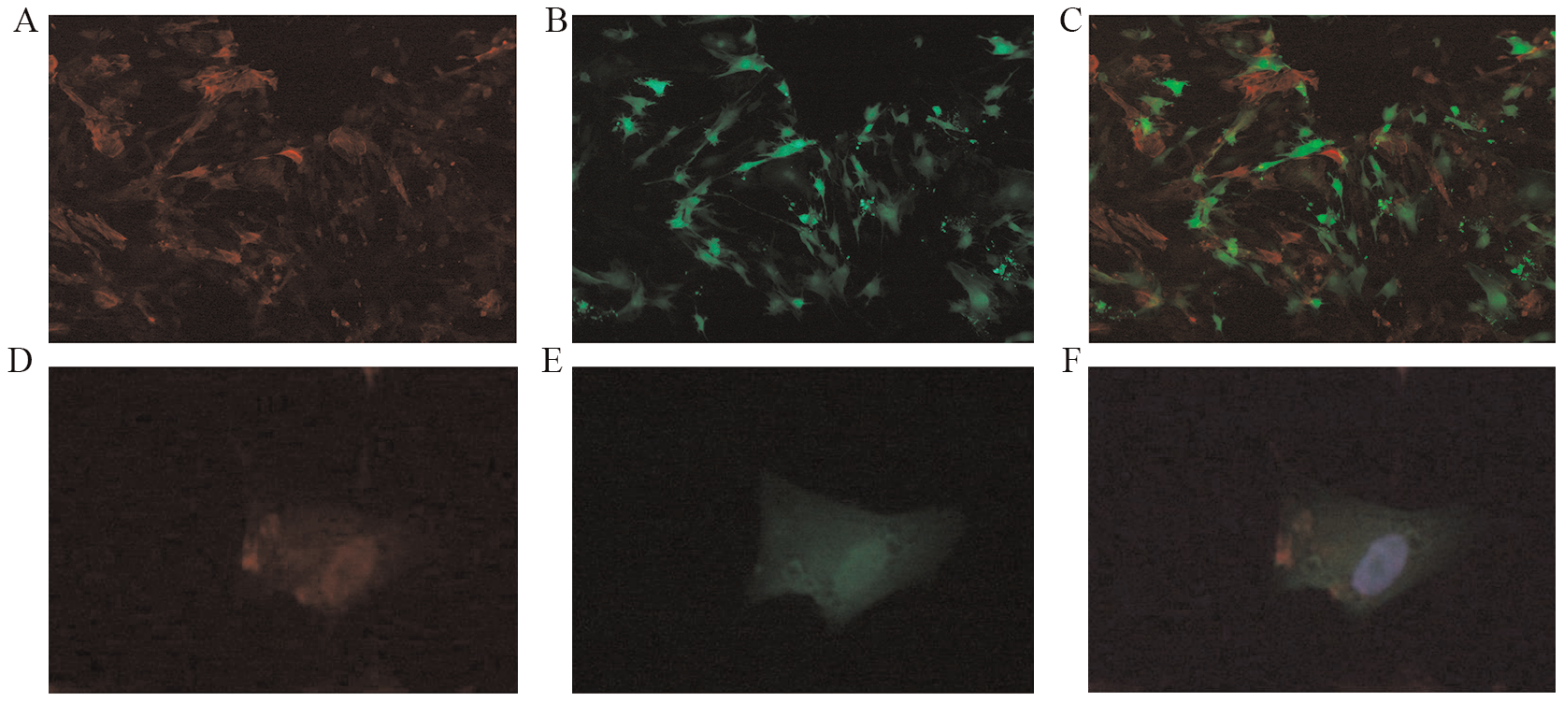
The expression of troponin T-CY3 in MSCs co-cultured with CMs. Co-culture of CMs (with positive staining for troponin T-CY3, red) (A) and EGFP+MSCs (green) (B) and CMs (with positive staining for troponin T-CY3, red) at a 1∶1 ratio (C). Representative EGFP+MSC (green) (E) is positively stained for troponin T-CY3 (red) (D) as shown in the merged cell image of D&E (F). Note: nuclei are stained with DAPI (blue).

After 2–3 days of the co-culture of EGFP+MSCs with CMs, morphological changes were observed in MSCs. In particularly, the long spindles of these MSCs gradually shortened and thickened in shape, and the extension of pseudopodia was observed. Fluorescence microscopy revealed that after 4–5 days of co-culture, a subset of EGFP+MSCs expressed cTnT (Figure 4). The co-culture was terminated after 12 days, and no spontaneous beating was observed in these EGFP+MSCs.

### 5. The expression of HDAC1 mRNA

**Figure 5:**
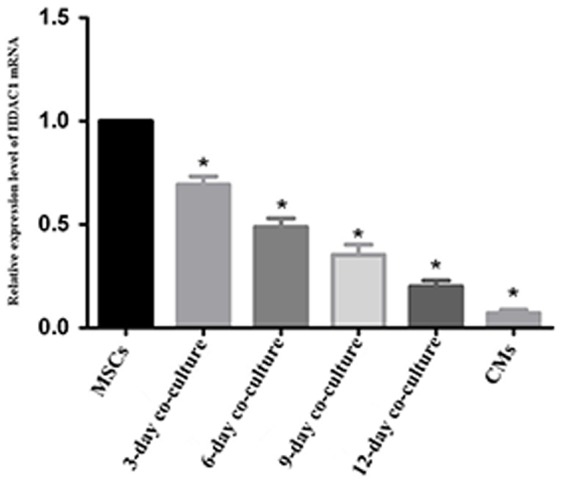
The relative HDAC1 mRNA levels. The HDAC1 mRNA levels in EGFP+MSCs co-cultured with CMs at different time points, in CMs without co-culture, and in MSCs without co-culture (MSCs, which is defined as 1 for its HDAC1 mRNA level). Note: n = 6, P<0.05, compared with that in MSCs group.

HDAC1 mRNA could be found in cells from all experimental groups. The HDAC1 mRNA expression in MSCs was significantly higher than that in either the CM group or the co-culture groups. In particularly, the 3-day co-culture, 6-day co-culture, 9-day co-culture, 12-day co-culture, and CM groups exhibited 0.69±0.04, 0.49±0.04, 0.35±0.05, 0.20±0.02, and 0.07±0.02 of the HDAC1 mRNA expression respectively compared with that in MSC group without co-culture, which was defined as 1, (p<0.05 compared with MSC group without co-culture). Approximately, a 14-fold higher HDAC1 mRNA expression than that in CMs was observed in MSCs without co-culture (Figures 5).

### 6. HDAC1 protein expression

**Figure 6:**
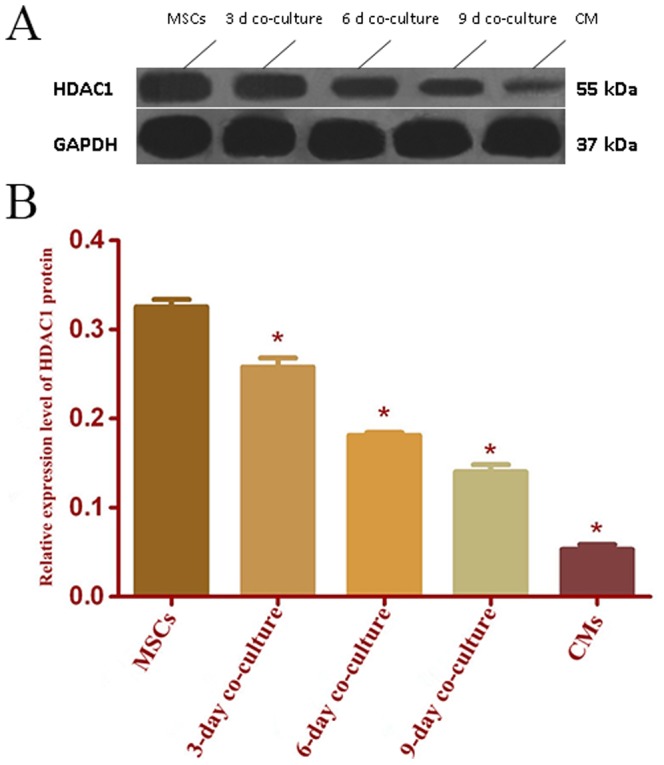
The HDAC1 protein levels. The HDAC1 protein levels in EGFP+MSCs co-cultured with CMs at different time points, in CMs without co-culture, and in MSCs without co-culture (MSCs). A. Representative western blots from different groups. B The HDAC1 protein levels from different groups. Note: n = 6, P<0.05, compared with that in MSCs group.

HDAC1 protein levels in EGFP+MSCs at 3, 6, and 9 days of co-culture with CMs were significantly lower than that in the control group (MSC group without co-culture; p<0.05). It should be noted that the HDAC1 protein level in control MSCs was approximately eight-fold higher than that in CMs (Figure 6).

## Discussion

This study demonstrated that MSCs could differentiate into CM phenotypes in a myocardial microenvironment. The expression of HDAC1 in MSCs was much higher than that in CMs.

However, when the MSCs co-cultured with CMs, the expression of HDAC1 was significantly decreased in a time-dependent manner. The decreased expression of HDAC1 was accompanied by the increased cardiac cell differentiation of MSCs as shown by the increased expression of cTnT. In addition, HDAC1 expression remained at low levels in CMs during the co-culture. The results suggest that HDAC1 has a negative correlation with cardiac cell differentiation from MSCs under the myocardial microenvironment.

Feng et al. reported that after 7 days of treatment with suberoylanilide hydroxamic acid (SAHA), an HDAC inhibitor, rat MSCs exhibited a SAHA dose-dependent increases in the mRNA expression levels of the CM-specific genes GATA4, Nkx2.5, and Mef2c [5]. The mRNA expression levels of these genes could be up to 15-fold higher than the corresponding expression levels in an untreated group. In addition, Muscari et al. demonstrated that in co-cultures of GFP-MSCs and CMs, treatment with difluoromethylornithine (DFMO), a polyamine synthesis inhibitor, causes the expression of cardiac myosin light chain 2 (cMLC2) and cardiac troponin I (cTnI) in GFP-MSCs to increase by 300% compared with that in untreated control group [6]. These findings suggest that histone acetylation plays an important role in the differentiation of MSCs into CM-like cells and the inhibition of HDACs could be a feasible method for promoting the directed cardiac-cell differentiation of stem cells. In the current study, we identified for the first time that a HDAC subtype, HDAC1 was significantly decreased during the differentiation of MSCs into CM-like cells in a myocardial microenvironment. Our recent report has found that knockdown of the HDAC1 could promote the directed differentiation of MSCs into CMs (in press). It will be interested to know whether the reduction in HDAC1 expression could reduce its HDAC1 activity, increase the acetylation of HDAC1 substrates and finally promote the expression of myocardial differentiation genes under the myocardial microenvironment both *in vitro* and *in vivo*.

HDACs are involved in the regulation of transdifferentiation of various stem cells to diverse cell lineages. The HDAC inhibitors SAHA and trichostatin A (TSA) can promote the differentiation of MSCs into not only CM-like cells but also the cells of other lineages, such as liver cells [12], endothelial cells [13], chondrocytes [14], and fat cells [15]. In other words, these inhibitors fail to promote the directed differentiation of MSCs into particular cell lineages. Moreover, SAHA and TSA are non-specific HDAC inhibitors that could affect multiple HDAC subtypes and may thereby unbalance cellular equilibrium with respect to histone acetylation and deacetylation. Dovey et al. reported that the deletion of HDAC1 in particular promoted the differentiation of embryonic stem cells into CM-like cells and neuron-like cells [16]. Furthermore, Liu et al. observed that HDAC1 overexpression inhibited the differentiation of P19CL6 cells into CMs, whereas P19CL6 cells, in which HDAC1 had been knocked-out, exhibited an upregulation of the cardiac-specific gene NKx2-5 expression [17]. Therefore, the specific inhibition of HDAC1 expression in MSCs might be a feasible method of promoting the directed differentiation of MSCs into CMs. Further studies should be performed to determine the roles other HDAC subtypes in the directed differentiation of MSCs to CMs.

In summary, in this study we have identified that HDAC1 has a negative correlation with cardiac cell differentiation from MSCs under a myocardial microenvironment. HDAC1 might play an important role in the directed differentiation of MSCs into CMs in heart.
